# Integrated analysis of single-cell and bulk RNA-sequencing reveals tumor heterogeneity and a signature based on NK cell marker genes for predicting prognosis in hepatocellular carcinoma

**DOI:** 10.3389/fphar.2023.1200114

**Published:** 2023-06-14

**Authors:** Shuo Li, Hongbo Du, Da’nan Gan, Xiaoke Li, Xiaobin Zao, Yong’an Ye

**Affiliations:** ^1^ Department of Gastroenterology, Dongzhimen Hospital, Beijing University of Chinese Medicine, Beijing, China; ^2^ Institute of Liver Diseases, Beijing University of Chinese Medicine, Beijing, China; ^3^ Key Laboratory of Chinese Internal Medicine of Ministry of Education and Beijing, Dongzhimen Hospital, Beijing University of Chinese Medicine, Beijing, China

**Keywords:** natural killer cells, hepatocellular carcinoma, prognosis, immune microenvironment, ScRNA-seq

## Abstract

**Background:** Natural killer (NK) cells are a type of innate immune cell that recognize and eliminate tumor cells and infected cells, without prior sensitization or activation. Herein, we aimed to construct a predictive model based on NK cell-related genes for hepatocellular carcinoma (HCC) patients and assess the feasibility of utilizing this model for prognosis prediction.

**Methods:** Single-cell RNA-seq data were obtained from the Gene Expression Omnibus (GEO) database to identify marker genes of NK cells. Univariate Cox and lasso regression were performed to further establish a signature in the TCGA dataset. Subsequently, qPCR and immunohistochemistry (IHC) staining were employed to validate the expression levels of prognosis signature genes in HCC. The effectiveness of the model was further validated using two external cohorts from the GEO and ICGC datasets. Clinical characteristics, prognosis, tumor mutation burden, immune microenvironments, and biological function were compared for different genetic subtypes and risk groups. Finally, molecular docking was performed to evaluate the binding affinity between the hub gene and chemotherapeutic drugs.

**Results:** A total of 161 HCC-related NK cell marker genes (NKMGs) were identified, 28 of which were significantly associated with overall survival in HCC patients. Based on differences in gene expression characteristics, HCC patients were classified into three subtypes. Ten prognosis genes (KLRB1, CD7, LDB2, FCER1G, PFN1, FYN, ACTG1, PABPC1, CALM1, and RPS8) were screened to develop a prognosis model. The model not only demonstrated excellent predictive performance on the training dataset, but also were successfully validated on two independent external datasets. The risk scores derived from the model were shown to be an independent prognosis factor for HCC and were correlated with pathological severity. Moreover, qPCR and IHC staining confirmed that the expression of the prognosis genes was generally consistent with the results of the bioinformatic analysis. Finally, molecular docking revealed favorable binding energies between the hub gene ACTG1 and chemotherapeutic drugs.

**Conclusion:** In this study, we developed a model for predicting the prognosis of HCC based on NK cells. The utilization of NKMGs as innovative biomarkers showed promise in the prognosis assessment of HCC.

## Introduction

Hepatocellular carcinoma (HCC) is the most common type of liver cancer and a leading cause of cancer-related deaths worldwide (Llovet et al., 2016; Villanueva, 2019). The incidence of HCC has been increasing in recent years, and it is estimated that approximately 1 million will have been diagnosed by 2025 (Bray et al., 2018). HCC is a complex disease characterized by persistent inflammatory harm, cellular regeneration and death (Forner et al., 2018). Irregularities in genetic expression and the tumor microenvironment were the fundamental factors that promote cancer cell survival (Hanahan and Weinberg, 2011).

Natural killer (NK) cells, a subset of innate lymphocytes, were involved in the early defense against cancer and certain viral infections and also played a key role in the immune response against HCC (Vivier et al., 2008; Yu and Li, 2017). In the early stages of HCC, NK cells limited tumor growth and spreaded by mechanisms such as direct killing of tumor cells and secretion of toxic cytokines (Sun et al., 2015). However, the immunosuppressive tumor microenvironment in HCC might compromise NK cell function. With the development of HCC, tumor cells evaded NK cell surveillance and attack through various mechanisms, such as reducing the expression of NK cell activation receptors NKG2D and ULBP, or increasing the expression of inhibitory receptors (Mantovani et al., 2020). Therefore, clarifying the interplay between HCC and NK cells is critical for the development of effective immunotherapeutic strategies against this deadly disease.

In this study, we identified distinct genetic subtypes in order to unravel the tumor heterogeneity of HCC. Moreover, we developed a prognosis model based on NK cells. We aimed to demonstrate the value of NK cell-related genes for assessing the prognosis of HCC patients through a comprehensive analysis of genomic data and explored differences in tumor genetics and immune landscape in HCC.

## Materials and methods

### Data source and acquisition

The single cell (sc) transcriptome file of GSE146115 was downloaded from the Gene Expression Omnibus (GEO) database (http://www.ncbi.nlm.nih.gov/geo/). The training datasets (LIHC) were obtained from The Cancer Genome Atlas (TCGA; https://tcga-data.nci.nih.gov/tcga/). The validation datasets (GSE14520 and LIRI-JP) were downloaded from the GEO database and the International Cancer Genome Consortium (ICGC; http://www.icgc.org), respectively.

### Identification of NK cell marker genes by single cell RNA-seq analysis

To ensure the retention of high-quality scRNA-seq data, three filtering criteria were implemented on the raw data matrix for each cell. Specifically, only genes that exhibited expression in a minimum of five single cells were retained, cells expressing fewer than 100 genes were discarded, and cells with greater than 5% expression of mitochondrial genes were excluded from analysis. The Seurat R package (Stuart et al., 2019) was utilized to preprocess the single-cell transcriptome datasets based on its functions. The data were initially normalized using the NormalizeData function with a scale factor of 10,000 and the LogNormalize normalization method. Next, the top 1,500 most variable genes were identified using the FindVariableFeatures method. Principal component analysis (PCA) was performed using the RunPCA function, and statistically significant PCs were identified using the Jackstraw function based on the proportion of variance explained. Cell clustering was executed by using FindNeighbors and FindClusters functions with default parameters. Subsequently, t-distributed stochastic neighbor embedding (t-SNE) was performed using the RunTSNE function. The function FindAllMarkers was used to analyze differentially expressed genes (DEGs) between various cell types. For identifying marker genes for each cluster, an adjusted *p*-value <0.05 and |log2 (fold change) | >1 was utilized. For cluster annotation, a reference-based annotation was performed using reference data from the Human Primary Cell Atlas (Mabbott et al., 2013). Lastly, we used SingleR (Aran et al., 2019) to annotate the clustering outcomes acquired via Seurat.

### Consensus clustering analysis

The consensus clustering analysis was performed to investigate the heterogeneity of NKRG expression in HCC, using the ConsensusClusterPlus algorithm (Wilkerson and Hayes, 2010) to reclassify patients. To determine the optimal number of subtypes, the cumulative distribution function (CDF) and consensus matrices were used.

### Tumor mutation burden and immunogenomic landscape analysis

The analysis involved creating a waterfall plot of the mutation landscape using the R package “maftools” (Mayakonda et al., 2018), which highlighted the genes with the highest mutation frequency (Top 20). The frequencies of copy number variation (CNV) were also calculated, and the resulting data were presented in lollipop plots. Additionally, the “RCircos” package in R software was utilized to visualize the locations of these genes on the chromosomes. The “estimate” package was employed to compute the immune or stromal fraction of the tumor microenvironment (TME) using the ESTIMATE algorithm (Yoshihara et al., 2013). The CIBERSORT algorithm (http://cibersort.stanford.edu/) was utilized to evaluate the infiltration of immune cells. To determine the frequency of each immune cell type, ssGSEA analysis was performed, yielding ssGSEA scores (Hänzelmann et al., 2013).

### Pathway and function enrichment analysis

The R package “clusterProfiler” was used to perform Gene Ontology (GO) and Kyoto Encyclopedia of Genes and Genomes (KEGG) analysis (Yu et al., 2012). A *p*-value of <0.05 was considered to indicate significant enrichment.

### Cell culture

HCC cells (Huh7 and HepG2) and LO2 cells (as control cells) were obtained from Fubo Bio (Beijing, China) and maintained in Dulbecco’s modified eagle medium (DMEM) supplemented with 10% fetal bovine serum (FBS), 100 U/mL penicillin, and 100 μg/mL streptomycin. The 5 × 10^5^ LO2, HepG2, and Huh7 cells were seeded in 6-well plates at 37°C in 5% CO_2_ with saturated humidity.

### Quantitative real-time PCR

Total RNA was extracted from human tissues or cells using the TRIzol reagent (Invitrogen, CA, United States) according to the manufacturer’s instructions and was quantified using Nanodrop 2000 (Wilmington, DE, United States). Total RNA (1 mg) was used as the template for cDNA synthesis using the cDNA reverse transcription kit (Toyobo, Jan). The quantitative reverse transcription-polymerase chain reaction (qRT-PCR) assay was conducted using the Real-time PCR Detection System (Agilent Technologies, United States) with the SYBR Green Real-time PCR Master Mix (Toyobo, Jan). The primers used in this study are provided in Table S1, using GAPDH as an internal control gene. The experiments were performed in triplicate and repeated three times.

### Immunohistochemistry analysis

The protein-level expression was evaluated through immunohistochemistry (IHC) staining of tumor and normal clinical samples using the Human Protein Atlas database (HPA, http://www.proteinatlas.org). The HPA database provided photomicrographs of IHC staining in HCC and matching normal tissues, along with pathology and tissue sections.

### Construction and validation of prognosis signature based on NK cell marker genes

Limma (Ritchie et al., 2015) were used to identify differentially expressed genes between tumor and normal tissue. Univariate Cox regression analysis was performed to evaluate the prognostic value of NK cell marker genes for overall survival (OS) in the TCGA cohort, with genes having *p* < 0.05 deemed as prognosis genes. LASSO Cox proportional hazards regression was then employed using the “glmnet” package to assess the prognosis genes, with 10-fold cross-validation conducted to select the best model. A multivariate Cox regression analysis was carried out to identify the prognostic values of specific gene signatures, with the risk model constructed by a linear combination of the mRNA expression of genes and the relevant risk coefficient. The patients were classified into low- or high-risk groups based on the median cut-off value. The discrimination and calibration of the risk model were assessed using receiver operating characteristics (ROC) curves and calibration curves. To assess the model’s diagnostic value and applicability, the clinical impact curve (CIC) were performed by using the resample bootstrap method (bootstrap replications = 1,000). The continuous net reclassification improvement (NRI) and integrated discrimination improvement (IDI) were computed in order to evaluate the improvement and applicability of the new model in reclassification. Confidence intervals for NRI and IDI were generated with the bootstrap method with 1,000 replications. The 10-fold and 1000-time bootstrap resampling were used to assess the stability of the model.

### Protein-Protein interaction and molecular docking

Protein-Protein interaction (PPI) analysis were performed with STRING (http://string-db.org). The protein structures were downloaded from the Uniprot database (http://www.uniprot.org), and the drug structures were downloaded from the Pubchem database (https://pubchem.ncbi.nlm.nih.gov). Molecular docking and binding energies were calculated by SwissDock (http://www.swissdock.ch/docking). Interactions between protein and drug were analyzed using the Protein-Ligand Interaction Profiler (PLIP; https://plip-tool.biotec.tu-dresden.de/plip-web/plip/index). The visualization of the docking structure was performed using PyMol software (version 2.5.4).

### Statistical analysis

The analysis of data in this study was carried out using the R software (version 4.2.0) for statistical analysis, and the Sangerbox platform (Shen et al., 2022) for bioinformatics analysis. The Wilcoxon rank-sum test was utilized to compare variables that were not normally distributed, while the independent Student’s t-test was used to compare continuous variables between two groups. Categorical variable data were analyzed using the chi-squared test. Correlations were examined using the Pearson chi-square test. *P* < 0.05 was set as a significant threshold.

## Results

### Identification of HCC-related NK cell marker genes

**FIGURE 1:**
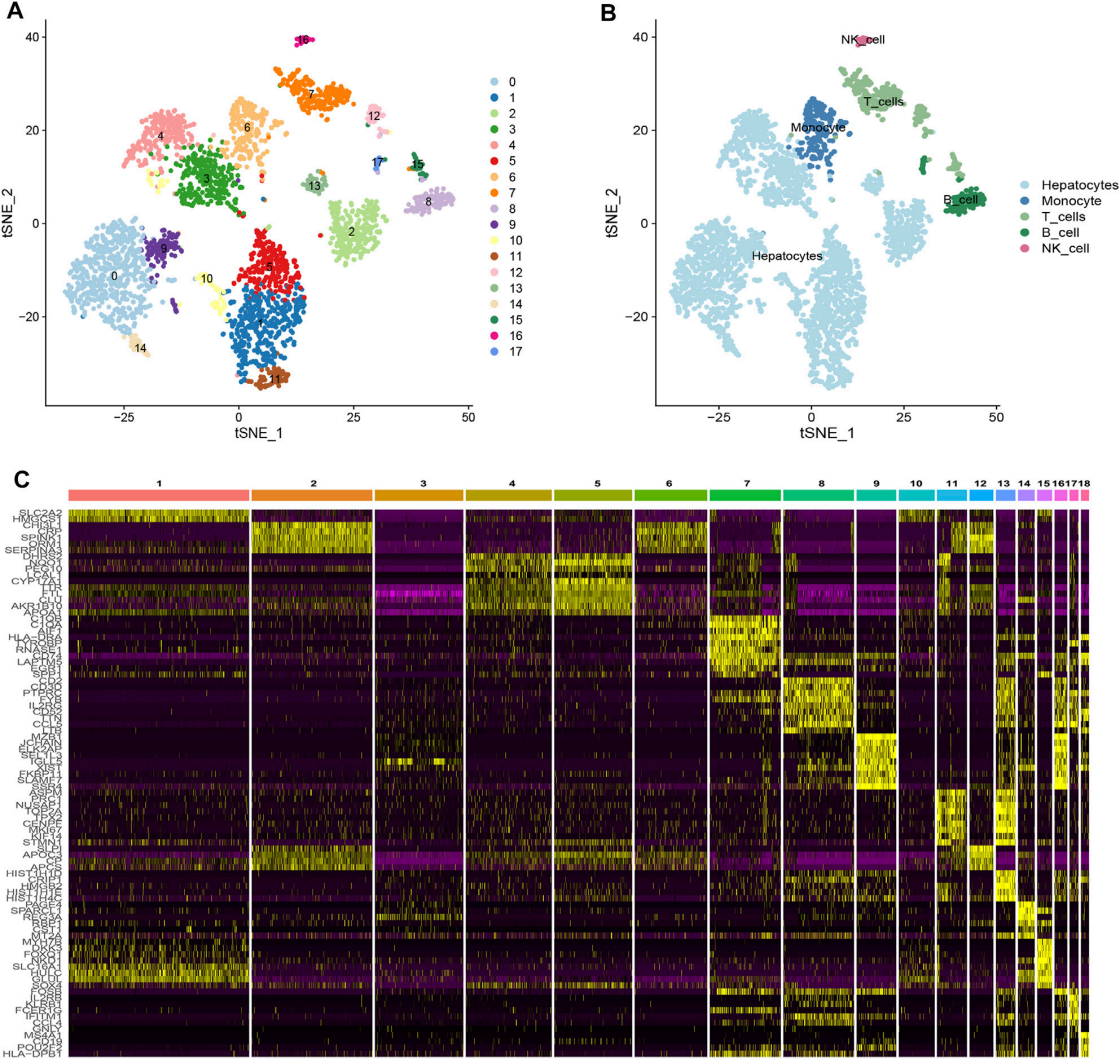
Single-cell RNA-sequencing analysis identified NK cell marker genes **(A)** tSNE clustering colored by groups. **(B)** The annotation of each cluster based on marker analysis. **(C)** Heatmap showing the top 5 marker genes in each cell cluster.

Based on scRNA-seq data from GSE146115, we obtained gene expression profiles of 3,200 cells from four HCC samples. We conducted PCA using the top 1,500 variable genes to reduce the dimensionality, and 18 cell clusters were identified (Figure 1A). Subsequently, the cells were annotated using a reference dataset from the Human Primary Cell Atlas and cells in the pink cluster were defined as NK cells (Figure 1B). 161 genes exhibited different expression profiles from other clusters and were defined as HCC-related NK cell marker genes (NKMG) (Figure 1C, Supplementary Table S2).

### Identification of three NK-related subtypes in HCC patients

**FIGURE 2:**
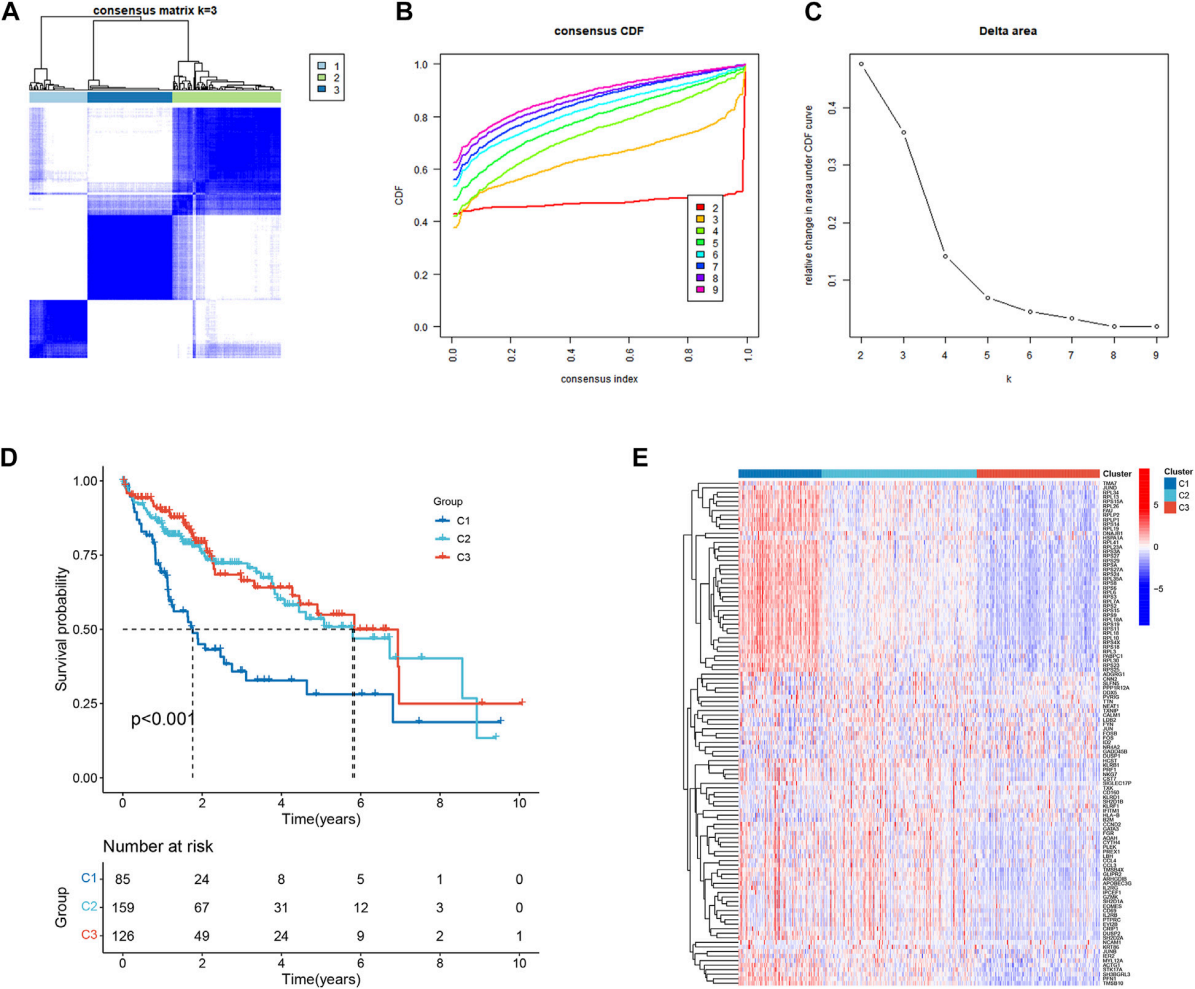
Identification and prognostic evaluation of three subtypes based on NKMGs in HCC. **(A)** Consensus clustering cumulative distribution function (CDF) for *k* = 2 to 9. **(B)** Relative change in the area under the CDF curve for *k* = 2 to 9. **(C)** HCC patients in the TCGA cohort were divided into four distinct clusters when *k* = 3. **(D)** K-M survival analysis of the OS status of HCC patients in three subtypes. **(E)** Heatmap of the differential NKMGs expression in three subtypes.

111 HCC-related NKMGs were significantly different between normal and HCC patients (Figure S1). After analysis of these genes expression characteristics by unsupervised clustering, patients with HCC in the TCGA cohort were divided into three subtypes (Figures 2A–C). Among them, we found a unique cluster 1 (C1), which had an extremely poor prognosis, and its median survival time was significantly lower than that of cluster 2 (C2) and cluster 3 (C3), almost one-third of theirs (*p* < 0.001, Figure 2D). Therefore, we further explored the differences in gene expression among the three subtypes and found that genes related to ribose-phosphate pyrophosphokinase (RPS) and prolactin (PRL) were significantly upregulated in C1 (Figure 2E). The functional enrichment, including GO and KEGG analysis, showed that the differential gene expression was mostly related to RNA transcription and protein synthesis, such as structural constituents of the ribosome, rRNA binding, SRP-dependent cotranslational protein targeting to the membrane, and nuclear-transcribed mRNA catabolic processes (Supplementary Figure S2).

### Tumor mutation burden and tumor immune microenvironments in three NK-related subtypes

**FIGURE 3:**
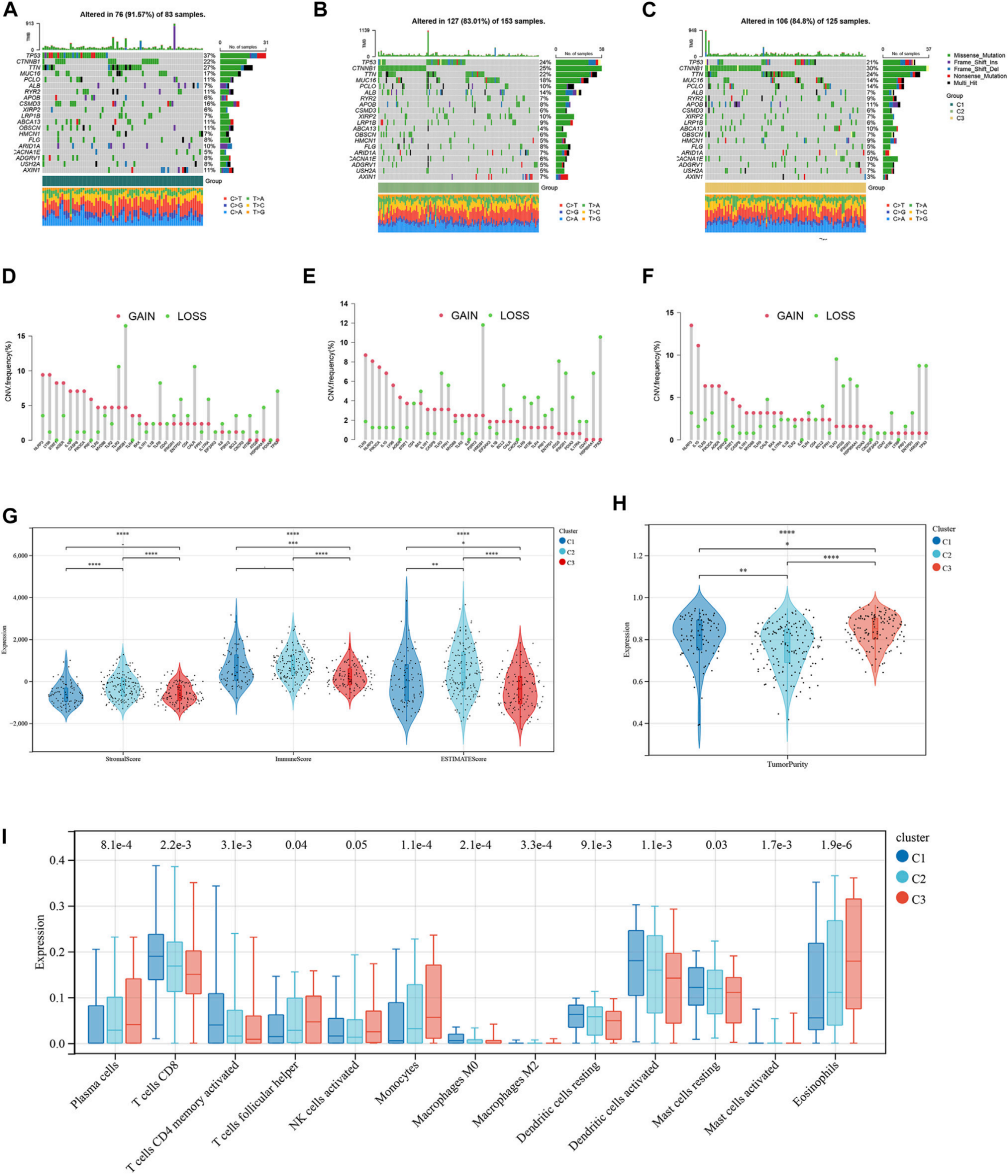
Tumor mutation burden and Immune microenvironment of three subtypes in HCC patients. **(A–C)** Waterfall maps of the somatic mutation landscape in three subtypes. **(D–F)** Lollipop diagrams of the copy number abnormalities indicates the degree of copy number loss (green) or gain (red). The stromal scores, immune scores **(G)**, and tumor purity **(H)** for three subtypes by Mann-Whitney *U*-test **(I)** The boxplot of immune infiltration cells between three subtypes of HCC. (*p < 0.05, **p < 0.01, ***p < 0.001, ****p < 0.0001).

The mutation frequencies of the HCC-related NKMGs (top 20) in HCC were initially identified in the three subtypes (Figures 3A–C). The mutations were mainly concentrated on four genes: TP53, CTNNBA, TTN, and MUC16. The mutation of CTNNB1 was the most important mutation event in C2 and C3 (accounting for 25% and 30%, respectively). However, the CTNNB1 mutation accounted for only 22% of the total mutational events in C1, which had the lowest percentage of CTNNB1 mutation events among the three subgroups. Compared to the other two subgroups, the mutation frequency of TP53 in C1 was the highest, reaching up to 37%. Most of the gain-of-function mutations were found in NLRP3 and LY96, whereas most of the loss-of-function mutations were located in NLRP3 and TLR3 (Figures 3D–F). Then, we explored the heterogeneity of immune microenvironments among different subtypes. Although there were differences between the groups, the results indicated that the tumor purity, stromal scores, and immune scores of C1 were mostly intermediate between those of C2 and C3 (Figures 3G, H). Subsequently, we employed the CIBERSORT algorithm to conduct further analysis of the immunological infiltration among different subgroups in order to observe the inherent differences in immune cell composition (Figure 3I; Supplementary Figure S2). The results showed that in C1, the expression levels of several immune cells were significantly upregulated, including T cells CD8, T cells CD4 memory activated, macrophages M0, dendritic cells activated, and mast cells resting. On the other hand, the expression levels of plasma cells, T cells follicular helper, monocytes, and eosinophils were significantly downregulated (*p* < 0.05).

### Identification and external validation of the NK cell prognosis signature in HCC

**FIGURE 4:**
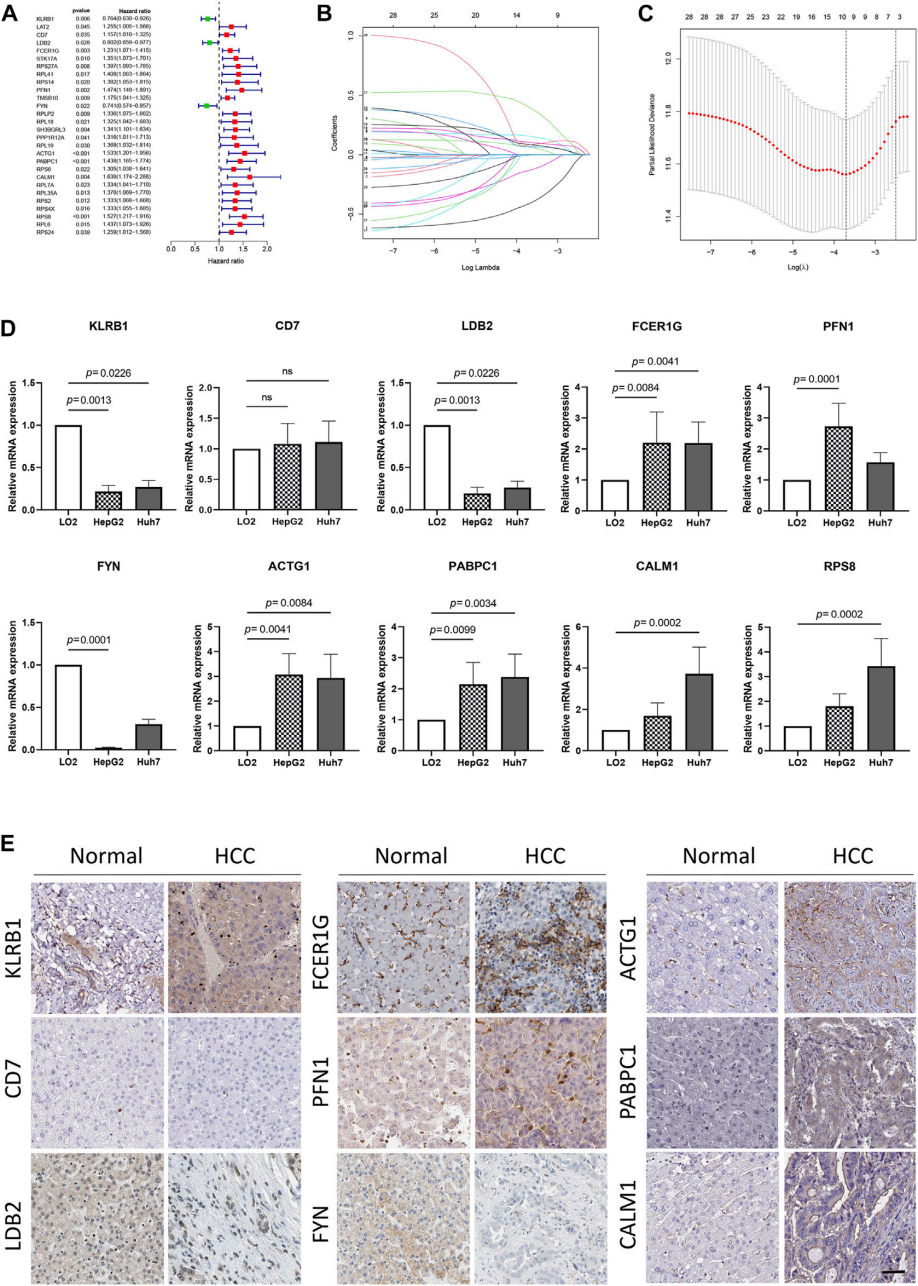
Identification and external validation of the HCC-related NK cell prognostic signature. **(A)** Forest plot based on univariate Cox analysis in the TCGA cohort. **(B)** LASSO coefficients of the NKMGs **(C)** Tenfold cross-validation for tuning parameter selection in the Least absolute shrinkage and selection operator (LASSO) model. Ten genes were selected by the LASSO Cox models **(D)**The expression levels of 10 genes in human normal liver cell line (LO2) and two HCC cell lines (HepG2 and Huh7) were examined by qRT-PCR. **(E)** The expression level of genes determined by immunohistochemistry in cancer tissues and normal tissues obtained from HPA datasets. Scale bar, 100 μm.

To develop a prognosis signature based on NKMGs, we first utilized the TCGA cohort as the training set to conduct a univariate Cox regression analysis. As a result, we identified 28 NK cell marker genes that were significantly associated with overall survival (OS) (Figure 4A). We subsequently performed LASSO Cox regression analysis, of which 10 were selected for inclusion in the prognosis signature, as shown in Figures 4B, C. These NKcell-related prognosis genes (NKPGs) were KLRB1, CD7, LDB2, FCER1G, PFN1, FYN, ACTG1, PABPC1, CALM1, and RPS8.

To further elucidate the relationship between prognosis genes and HCC, we conducted qPCR analysis on a human normal liver cell line (LO2) and two HCC cell lines (HepG2 and Huh7) (Figure 4D). The results demonstrated that the expression of FCER1G, PFN1, ACTG1, PABPC1, CALM1, and RPS8 was significantly upregulated in the liver cancer cells, whereas KLRB1, LDB2, and FYN exhibited the opposite trend. These findings were consistent with results obtained from the TCGA cohort, except for FCER1G (Supplementary Figure S2). While CD7 and FCER1G displayed an upward trend in HCC, there were no significant differences between the groups. Except for RPS8, which was not available in the HPA database, we explored the protein expression of other NKPGs in HCC tissues (Figure 4E). Compared with normal liver tissue, FCER1G, PFN1, ACTG1, PABPC1, and CALM1 were found to be highly expressed, while KLRB1, LDB2, and FYN were found to be lowly expressed in HCC.

### Establishment and validation of the HCC-related NK cell prognosis model

**FIGURE 5:**
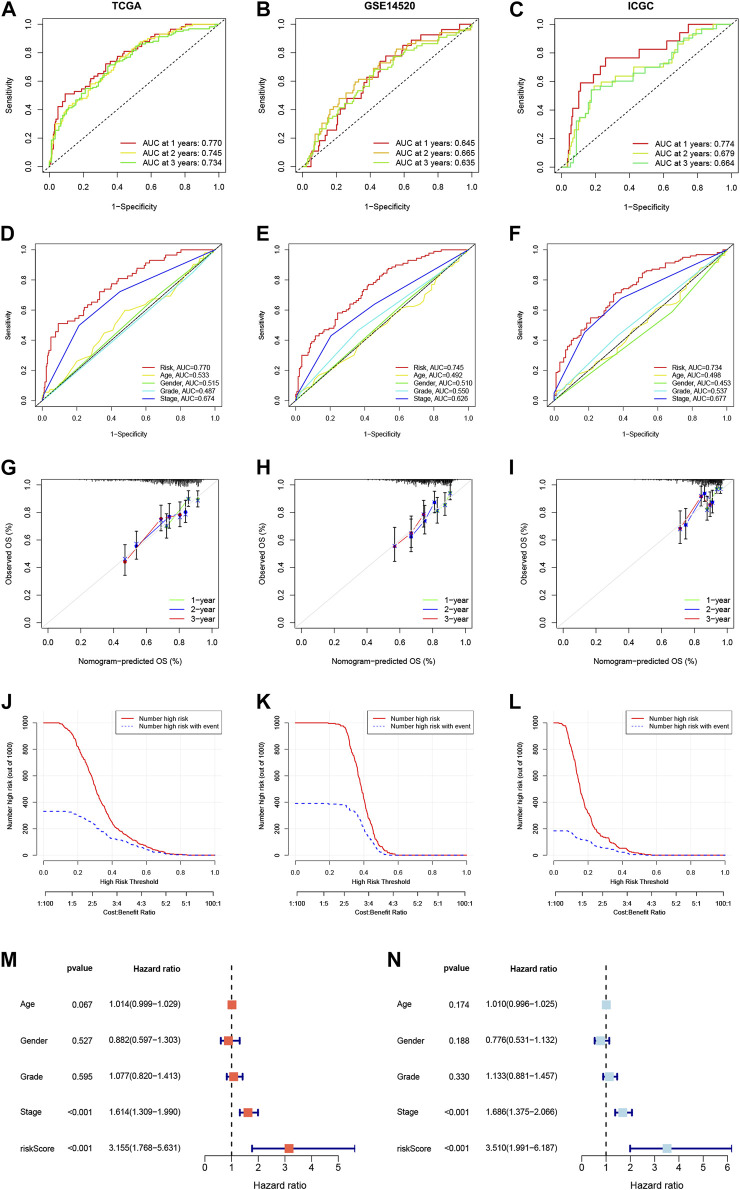
Establishment and validation of the HNK-10 models in HCC. Time-dependent receiver operating characteristic (ROC) curve analysis in the TCGA **(A)**, GEO **(B),** and ICGC **(C)** cohorts. ROC curves of clinical parameters in the TCGA **(D)**, GEO **(E),** and ICGC **(F)** cohorts. The calibration curves for 1-, 2-, and 3-year overall survival in the TCGA **(G)**, GEO **(H),** and ICGC **(I)** cohorts. Clinical impact curves for predicting OS in HCC patients in the TCGA **(J)**, GEO **(K),** and ICGC **(L)** cohorts. Univariate **(M)** and multivariate **(N)** Cox regression analysis of clinicopathological features.

We constructed a Prognosis model using the 10 NKPGs selected above and named it HNK-10. The risk score of each HCC patient was calculated as follows: Risk score = (−0.414 × KLRB1 expression) + (0.067 × CD7 expression) + (−0.003 × LDB2 expression) + (0.157 × FCER1G expression) + (0.068 × PFN1 expression) + (−0.133 × FYN expression) + (0.105 × ACTG1 expression) + (0.087 × PABPC1 expression) + (0.403 × CALM1 expression) + (0.083 × RPS8 expression). To validate the performance of the model, we conducted a time-dependent ROC analysis in three independent cohorts. In the TCGA cohort, the AUCs for 1-year, 2-year, and 3-year OS were 0.770, 0.745, and 0.734, respectively (Figure 5A). In the GSE14520 cohort, the corresponding AUCs were 0.645, 0.665, and 0.635 (Figure 5B). In the ICGC cohort, the AUCs were 0.774, 0.679, and 0.664, respectively (Figure 5C). The risk scores demonstrated superior discrimination compared to age, gender, tumor stage, and pathological grade, as evidenced by significantly higher AUC values (Figures 5D–F). The calibration curves showed a favorable level of concordance between the model predictions and the actual observed probabilities (Figures 5G–I). Moreover, the clinical impact curves indicated that the model had a positive impact on clinical decision-making, further supporting its efficacy (Figures 5J–L). As demonstrated in Figures 5M, N, univariate and multivariate Cox regression analysis revealed that the risk scores were independent predictors of OS compared to other clinical indications. The NRI and IDI showed that the HNK-10 model had better predictive accuracy than other clinical parameters (Supplementary Table S3). The 1000-time bootstrap accuracy was 70.68% and 10-fold accuracy was 71.18%, which showed good robustness of the HNK-10 model.

### The HNK-10 model had better discrimination for immune-related HCC patients

**FIGURE 6:**
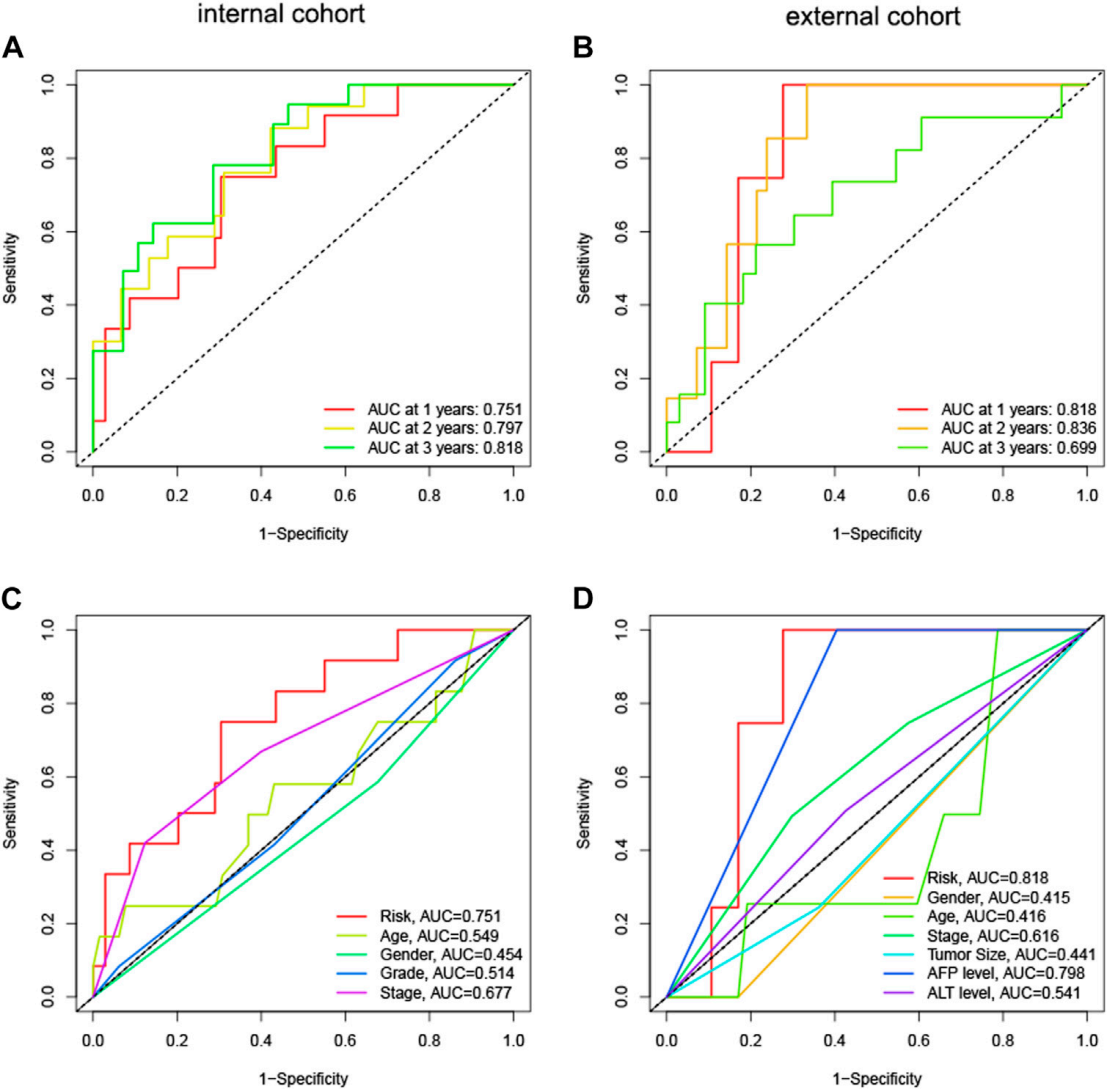
The discrimination of HNK-10 model for immune-related HCC patients. Time-dependent receiver operating characteristic (ROC) curve analysis in the internal **(A)** and external **(B)** cohorts. ROC curves of clinical parameters in the internal **(C)** and external **(D)** cohorts.

We computed immune scores for patients using the ESTIMATE algorithm. Patients with immune scores exceeding 1,000 were classified as immune-related patients. They were put into the model as the internal (TCGA) and external (GEO) cohorts. In the internal cohort, the AUCs for 1-year, 2-year, and 3-year OS were 0.751, 0.797, and 0.818, respectively (Figure 6A). In the external cohort, the AUCs for 1-year, 2-year, and 3-year OS were 0.818, 0.836, and 0.699 (Figure 6B). Compared to other clinical parameters, risk scores demonstrated better discrimination (Figures 6C, D). These results suggested that the HNK-10 model had more accurate predictive power in immune-related HCC patients compared to the full cohort of patients.

### The relationship between risk score, prognosis and pathological state

**FIGURE 7:**
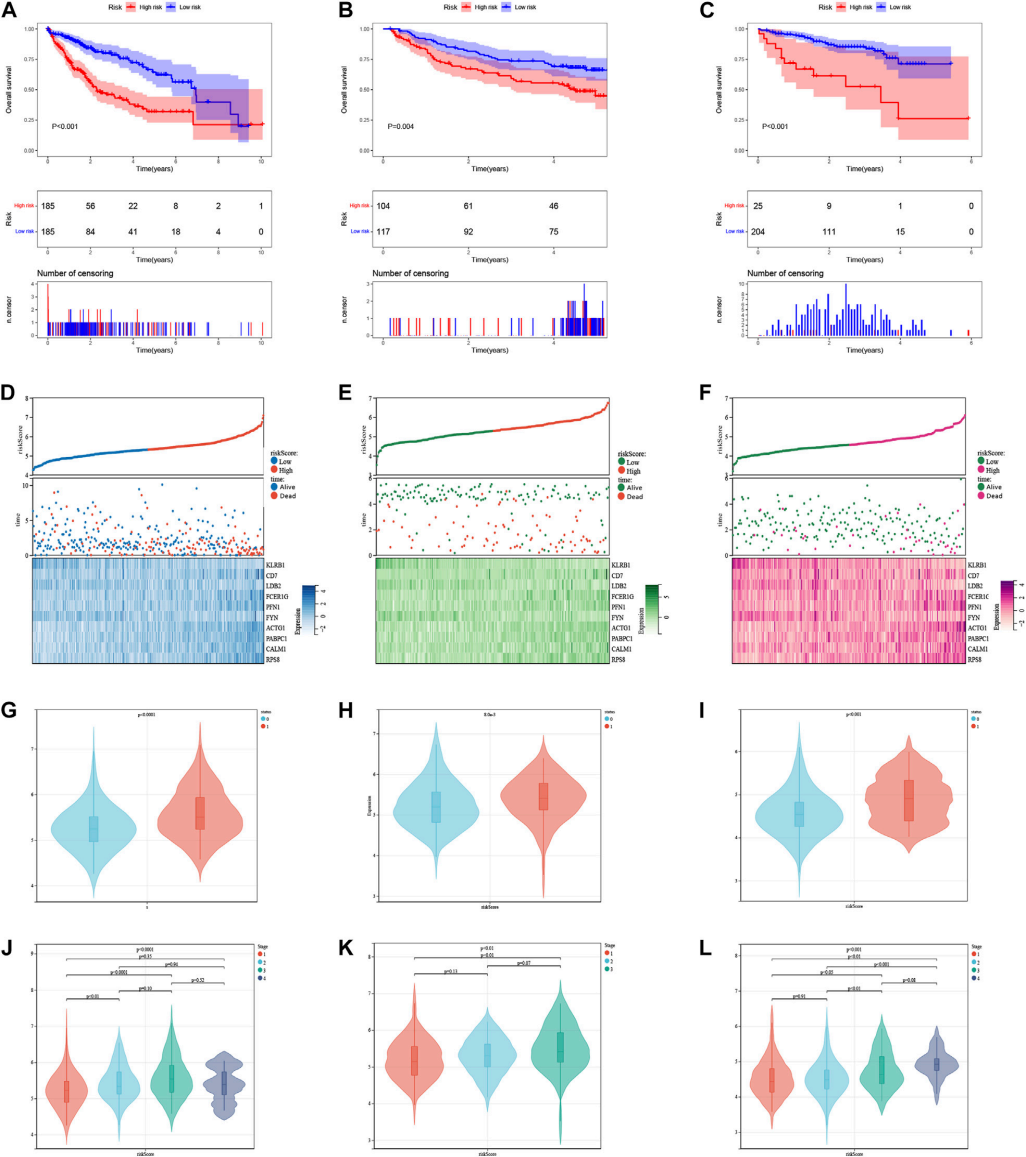
The risk score was related to HCC prognosis and pathological state. **(A)** K-M survival analysis of the ICDRGs risk model in the TCGA **(A)**, GEO **(B),** and ICGC **(C)** cohorts for HCC patients. **(B)** Risk triple plots, including risk dispersion plots, survival time scatter plots, and heatmaps of model gene expression in the TCGA **(D)**, GEO **(E),** and ICGC **(F)** cohorts. Boxplots of risk scores in HCC patients with different status of survival **(G–I)** and stages **(J–L)**. Status: 0 = alive, 1 = death.

The median risk score was 5.315, which divided the patient population into low-risk (*n* = 185) and high-risk (*n* = 185) groups. The KM survival curves revealed significantly lower OS in the high-risk group compared to the low-risk group in three cohorts (*p* < 0.05, Figures 7A–C). The relationship between risk scores and vital status among HCC patients was illustrated using scatter plots and risk curves (Figures 7D–F). Additionally, higher risk scores were significantly associated with poor survival status (Figures 7G–I) and advanced tumor stages (Figures 7J–L) across all three cohorts.

### Tumor mutation burden and microenvironment landscape in different HCC risk groups

**FIGURE 8:**
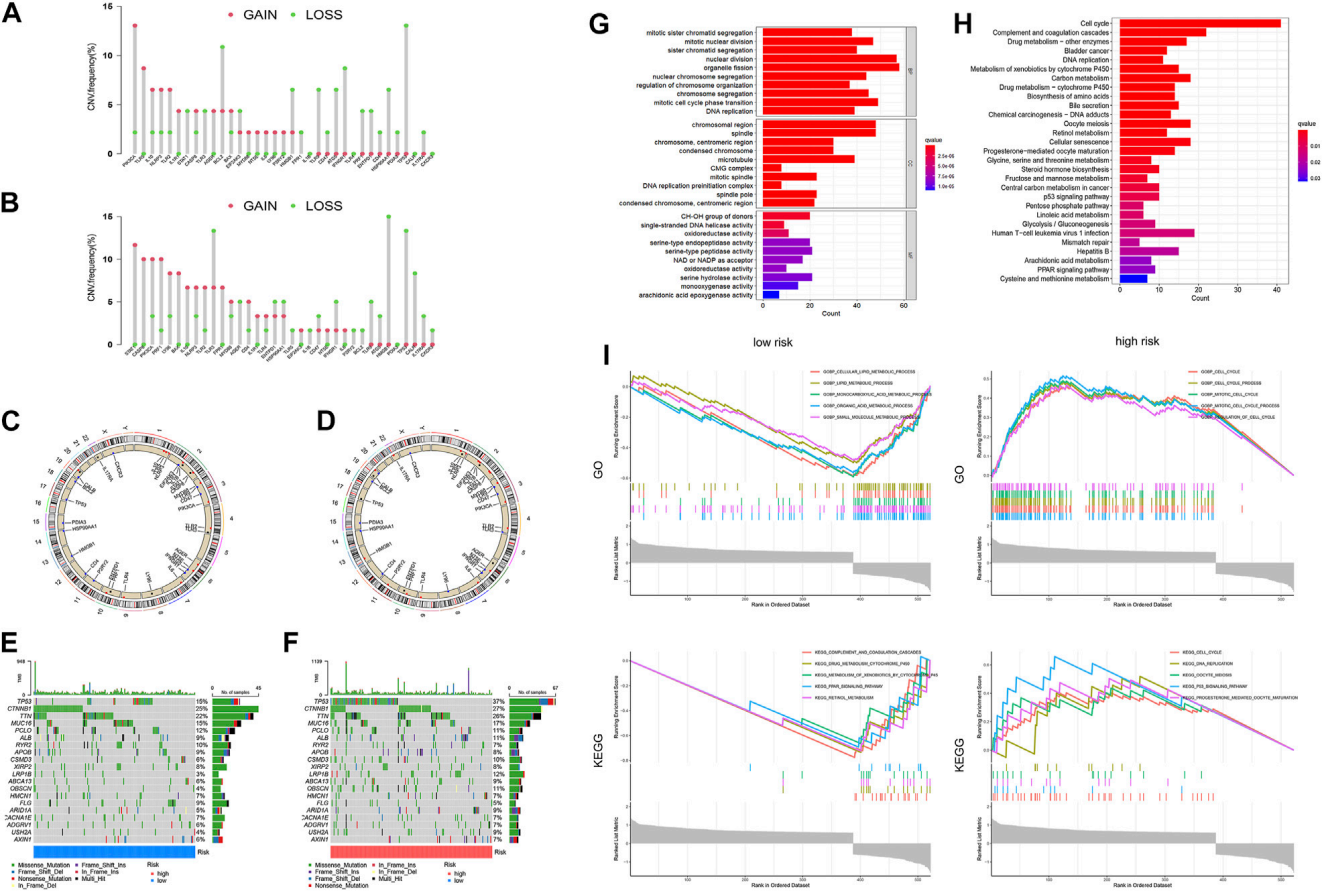
Differences in Tumor microenvironment and biological functions between different risk groups. The lollipop diagrams of the copy number abnormalities in the low-**(A)** and high-**(B)** risk groups (loss for green; gain for red). Circus plots of the chromosome distributions of selected genes in the low-**(C)** and high-**(D)** risk groups. Waterfall maps of the somatic mutation landscape in the low-**(E)** and high-**(F)** risk groups. The bar plot of the GO **(G)** and KEGG **(H)** pathways enrichment. **(I)**The gene set enrichment analysis (GSEA) for GO and KEGG for high-risk and low-risk groups of HCC patients.

In the low-risk group, most of the gain-of-function mutations were observed in PIK3CA, while most loss-of-function mutations were found in TP53 (Figure 8A). In the high-risk group, most of the gain-of-function mutations were observed in STAT1, while most loss-of-function mutations were located in HGMB1 (Figure 8B). The chromosome locations of these gene mutations were shown in Figures 8C, D. Furthermore, we compared the mutation profiles of the top 20 genes in different HCC subtypes (Figures 8E, F). Notably, the high-risk group had a higher frequency of TP53 mutations (37%) compared to the low-risk group (15%).

### Differences in biological functions between different risk groups of HCC

In order to identify the molecular mechanisms regulating prognosis, we further identified the key 2170 DEGs in high- and low-risk groups (Supplementary Figure S5) and then performed GO and KEGG pathway enrichment analysis on the above DEGs. Based on the results of the GO analysis, the DEGs were predominantly enriched in pathways related to nuclear division, organelle fission, mitotic cell cycle phase transition, chromosomal region and spindle (Figure 8G). Based on the results of the KEGG analysis, the DEGs were predominantly enriched in pathways related to cell cycle, complement and coagulation cascades, drug metabolism, DNA replication, and metabolism of xenobiotics by cytochrome P450 (Figure 8H). We additionally conducted GSEA analysis, as demonstrated in Figure 8I. Based on the results of GO enrichment analysis, biological processes highly associated with cell cycle process, mitotic cell cycle process, and regulation of cell cycle in the high-risk group. Cellular lipid metabolic process, lipid metabolic process, monocarboxylic acid metabolic process, organic acid metabolic process, and small molecule metabolic process were enriched in the low-risk groups. Based on the results of KEGG enrichment analysis, biological processes highly associated with cell cycle, DNA replication, oocyte meiosis, p53 signaling pathway, and progesterone mediated oocyte maturation in the high-risk group. Complement and coagulation cascades, drug metabolism cytochrome p450, metabolism of xenobiotics by cytochrome p450, retinol metabolism and steroid hormone biosynthesis were enriched in the low-risk groups.

### The docking conformation and interaction force analysis of HNK-10 hub gene

**FIGURE 9:**
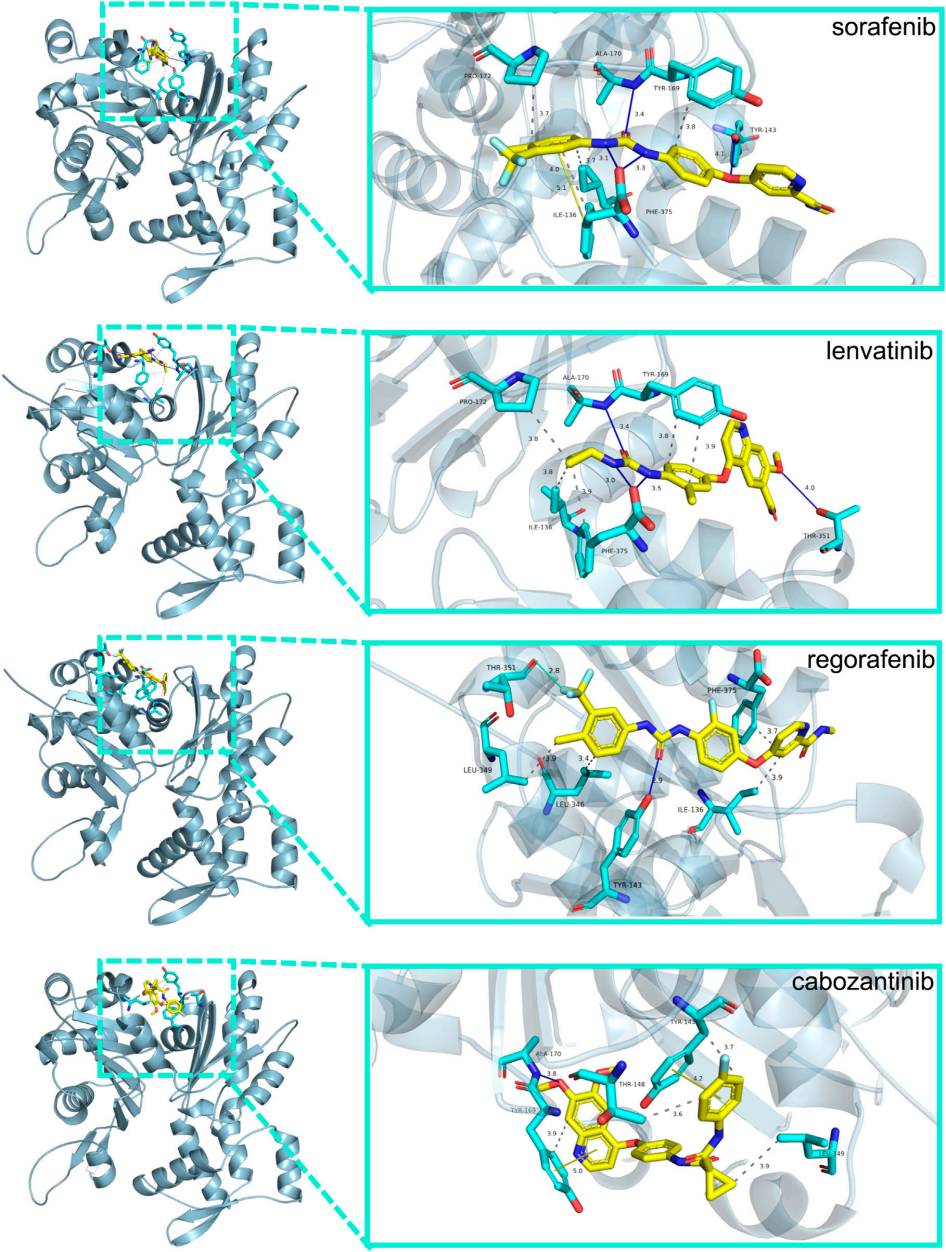
The docking conformation and interaction force analysis between ACTG1 and sorafenib, lenvatinib, regorafenib and cabozantinib. Color symbols: yellow sticks for drug molecules, cyan sticks for amino acid residues, blue lines for hydrogen bonding, green lines for halogen bonding, yellow lines for π-Stacking, and gray dashed lines for hydrophobic interaction.

We used molecular docking to explore the role of NKPGs in chemotherapy. We first performed PPI analysis on 10 prognosis genes. Among them, ACTG1, which had the highest degree, was identified as the hub gene (Supplementary Figure S6). Molecular docking of ATCG1 was performed with the main chemotherapeutic agents [sorafenib, lenvatinib, regorafenib, and cabozantinib (Llovet et al., 2021)] used in first- and second-line clinical practice. Docking conformation and interaction force analysis of ACTG1 with four mainstream chemotherapeutic agents were shown in Figure 9. The results indicated that sorafenib forms four hydrophobic interactions, four halogen bonds, and 1 π-Stacking with amino acid residues of ACTG1, with a binding energy of −9.01 kcal/mol. Lenvatinib forms five hydrophobic interactions and four hydrogen bonds with amino acid residues of ACTG1, with a binding energy of −8.84 kcal/mol. Regorafenib forms four hydrophobic interactions, one hydrogen bond, and one halogen bond with the amino acid residues of ACTG1, with a binding energy of −8.43 kcal/mol. Cabozantinib forms 4 hydrophobic interactions, 1 hydrogen bond, and 2 π-Stacking with amino acid residues of ACTG1, with a binding energy of −8.70 kcal/mol.

## Discussion

The emergence of HCC was gradual and unnoticeable, and the initial symptoms were not typical and posed difficulty in diagnosis. To address this challenge, our study aimed to develop an NK-related prognosis model consisting of 10 genes to predict the prognosis of HCC patients effectively. The results also highlighted the heterogeneity of the tumor immune microenvironment in different subtypes and risk groups, which might help to elucidating the immunological and biological mechanisms of poor prognosis.

According to the NKMGs signatures, we identified three distinct subtypes. The subtypes showed tumor heterogeneity mainly in terms of extensive genomic alterations and immune microenvironment. Notably, the clusters with the poorest prognosis had higher expression levels of the RPS and PRL protein families. The RPS protein family refers to the S family of ribosomal proteins on the ribosome, which are involved in the structure and function of the ribosome. Several studies have shown that members of the RPS family promote the development and metastasis of hepatocellular carcinoma mainly by increasing the proliferation and invasive ability of hepatocellular carcinoma cells (Calvisi et al., 2011; Guo et al., 2018). The PRL protein family is a group of proteins that are involved in a variety of physiological processes, including lactation, reproduction, and immune function. Some PRL family members mediated the phosphorylation of FAK, thus promoting the progression of hepatocellular carcinoma (Zhou et al., 2020). The results suggested that tumor cells might evade immune surveillance by NK cells by regulating ribosome synthesis and functional protein secretion to metastasize and invade tissues.

Next, we explored the heterogeneity of immune microenvironments among different subtypes. By analyzing the immune infiltration of the three subtypes, we found significant changes in the distribution and number of immune cells. As for the immune score, stromal score, and tumor purity in the three subtypes, interestingly, the subtype with the worst prognosis had scores almost in between the other two subtypes. This indicated that alterations of the immune microenvironment caused by differences in gene expression might be a key factor affecting prognosis. However, the ratio of immune cells to stromal cells might not be the basic reason for prognosis differences, but rather the ratio of various immune cells.

To better apply NKMGs to the clinical diagnosis of HCC, we screened 10 prognosis genes (KLRB1, CD7, LDB2, FCER1G, PFN1, FYN, ACTG1, PABPC1, CALM1, and RPS8). *In vitro* cell experiments and IHC, the expression of these genes in HCC was validated. Subsequently, we developed a new prognosis model (HNK-10). In the training and two external validation cohorts, the HNK-10 model demonstrated steady and reliable predictive performance. More importantly, the risk score calculated based on the model is an independent prognosis risk factor for HCC patients. Interestingly, we found that the HNK-10 model had better discrimination for immune-related HCC patients, which suggested that our model might be more applicable for the prognosis diagnosis of immune-related patients.

KLRB1 was the gene encoding CD161, which has been shown to inhibit the cytotoxicity of NK cells. Thus, KLRB1 downregulates the inhibitory molecule CD161 and enhances the ability of NK cells to kill infected or transformed cells (Aldemir et al., 2005). CD7 was a transmembrane glycoprotein normally expressed by the majority of peripheral T-cells and NK cells and their precursors, serving as a co-stimulatory protein aiding T-cell activation and interaction with other immune subsets (Rabinowich et al., 1994; Gomes-Silva et al., 2017). The stimulation of plate-bound anti-CD7 induced the production of IFN-γ and the proliferation of NK cells (Milush et al., 2009). Yu et al. (2017) identified a potential role for LDB2 in the pathogenesis of HCC, as significant downregulation of LDB2 was observed in most HCC samples and the ability of LDB2 to inhibit the proliferation and migration of HCC cells. While the function of FCER1G in HCC is not yet fully understood, a study by Dong et al. (2022) suggested that FCER1G was associated with macrophage infiltration and played a role in promoting unfavorable prognosis by affecting tumor immunity in clear cell renal cell carcinoma. PFN1 was mainly responsible for the polymerization of actin filaments and responds to extracellular signals, which were associated with cell proliferation and motility (Witke, 2004). Xie et al. (2018) suggested that PFN1 was a risk factor for poor prognosis in HCC. This was consistent with our findings. However, PFN1 was considered to be a suppressor molecule in breast cancer and its deletion leads to enhanced motility and invasiveness of breast cancer cells (Zou et al., 2007). Previous studies suggested that overexpression of PFN1 upregulated PTEN and inhibited AKT activation in breast cancer cells (Das et al., 2009). This difference may be due to the heterogeneity of the tumor. It was shown that mice overexpressing FYN had significantly reduced tumor volume and weight, suggesting that FYN significantly inhibits malignancy and promotes apoptosis of tumor cells (Huang et al., 2022). ACTG1 promoted HCC proliferation by regulating the cell cycle through downregulation of cell cycle proteins and cell cycle protein-dependent kinases, as well as inhibiting apoptosis through extra-mitochondrial pathways (Yan et al., 2019). High expression of PABPC1 was associated with low overall survival in HCC and was an independent prognosis factor in HCC (YuFeng and Ming, 2020). CALM1 was identified as one of the overexpressed genes in various cancers, mainly associated with cell proliferation, programmed cell death, and autophagy (Adeola et al., 2016; Zamanian Azodi et al., 2018; Zhang et al., 2018; Liu et al., 2021). RPS8 was confirmed to be highly expressed in alcohol-related HCC (Bi et al., 2020).

Using the HNK-10 model, we calculated the risk score for each HCC patient and found it to be an independent risk factor for poor prognosis. In addition, multiple bioinformatics analyses showed significant differences in gene mutation and immunological status between the high- and low-risk groups. Next, we conducted enrichment analysis on differentially expressed genes between high- and low-risk groups, and found that the main pathways were concentrated in the cell cycle, such as cell division and DNA replication. NK cells recognized and killed certain abnormal cells, including those with excessive DNA damage or in preparation for division during the cell cycle (Wu et al., 2020). In this case, NK cells destroy these abnormal cells by releasing cytotoxins or inducing apoptosis, thereby maintaining immune balance and homeostasis in the body. Interestingly, we also found that another part of the pathways was enriched in metabolism, especially drug metabolism in KEGG enrichment, which caught our attention. This suggested that NKMGs may not only play a role in immune regulation, but may also be effective in chemical drug therapy, which becoming a bridge between immunotherapy and chemotherapy. Therefore, we identified the hub gene ACTG1 among 10 prognosis genes through PPI analysis, and conducted molecular docking with four chemotherapy drugs in clinical practice. The binding energies were all less than −8.0 kcal/mol, indicating that the active ingredients have strong affinity with the target, and were stably bound to the target protein of ACTG1.

Our study still had some limitations. First, since this study was a retrospective investigation, prospective studies with real-world analysis are necessary to validate the application of this strategy. Second, our experimental validation was cell-based *in vitro*. If these results could be validated in animal models or clinical patients samples, it might increase the persuasiveness of this study.

In conclusion, this study concluded by identifying NKMGs in HCC patients, developing and validating a model for predicting the prognosis of HCC patients, which exhibited robust predictive capabilities. We also explored the differences of genetic mutations and immunological microenvironment to observe tumor heterogeneity from the perspective of NK cells. Our study contributed to a better understanding of the role of NK cells in HCC progression and provided evidence for NK-related genes as innovative predictors of prognosis in HCC.

## Data Availability

Publicly available datasets were analyzed in this study. This data can be found here: TCGA database (https://portal.gdc.cancer.gov/); Gene Expression Omnibus (GEO) database (http://www.ncbi.nlm.nih.gov/geo/); ICGC database (https://dcc.icgc.org/) and Human Protein Atlas database (http://www.proteinatlas.org).
