# Supplementary material for: Integrated analysis of single-cell and bulk RNA-sequencing reveals tumor heterogeneity and a signature based on NK cell marker genes for predicting prognosis in hepatocellular carcinoma
Source: Front Pharmacol. 2023 Jun 14;14:1200114. doi: 10.3389/fphar.2023.1200114 (PMC10307919; doi:10.3389/fphar.2023.1200114)
Supplement: Supplementary file 1 [file DataSheet1.docx]

**Supplementary Table**

**Table S1** The primers used in this study.

| Gene | Primer | Sequence |
| --- | --- | --- |
| KLRB1 | F | TGGCATCAATTTGCCCTGAAA |
|  | R | TCCAAGGGTTGACAGTGTGAG |
| CD7 | F | ACCTGACTATCACCATGCACC |
|  | R | CGGAGCCGTAGACATTGACC |
| LDB2 | F | AGGATAGTGACAACCTCTGGTG |
|  | R | GAGGGTCCTGCCGATAGTGTA |
| FCER1G | F | AGCAGTGGTCTTGCTCTTACT |
|  | R | TGCCTTTCGCACTTGGATCTT |
| PFN1 | F | ATGGATCTTCGTACCAAGAGCA |
|  | R | CCATCAGCAGGACTAGCGTC |
| FYN | F | TGGAGGTGTGAACTCTTCGTC |
|  | R | TCTGTCCGTGCTTCATAGTCA |
| ACTG1 | F | CCGAGCCGTGTTTCCTTCC |
|  | R | GCCATGCTCAATGGGGTACT |
| PABPC1 | F | CAGGCTCACCTCACTAACCAG |
|  | R | GGTAGGGGTTGATTACAGGGT |
| CALM1 | F | AAATCCGTGAGGCATTCCGAG |
|  | R | CATGACGTGACGTAGTTCTGC |
| RPS8 | F | GCTCAGAGTGTTGTACTCGTAAA |
|  | R | AGCACGATGCAATTCTTCACC |

F = Forward, R = Reverse.

**Table S2** 161 genes defined as HCC-related NK cell marker genes.

| KLRC1 | HCST | RPS11 | RPSA |
| --- | --- | --- | --- |
| SH2D1B | RPS27 | GADD45B | RPS6 |
| IL2RB | IPCEF1 | 7-Sep | RPS15 |
| KLRF1 | TMSB4X | JUNB | RPL10 |
| SIGLEC17P | ALOX5AP | RPL23A | GMFG |
| TXK | SLC2A3 | TMSB10 | SH2D2A |
| NCAM1 | STK17A | CYTH4 | DNAJB1 |
| CD247 | PREX1 | CNN2 | CALM1 |
| KLRB1 | DOCK10 | HLA-B | CELF2 |
| CTSW | AOAH | FYN | RPL7A |
| KRT86 | GZMB | CCND2 | RPS3A |
| MATK | IFITM2 | RPLP2 | CRIP1 |
| NKG7 | WIPF1 | FGD3 | NFKBIA |
| LAT2 | ETS1 | RPL18 | RPL13 |
| CD160 | DOK2 | KLRD1 | GATA3 |
| CD7 | ARHGDIB | RPS18 | RPL3 |
| LDB2 | ADGRG1 | B2M | PLEK |
| GZMA | CCL5 | SH3BGRL3 | RPL35A |
| GZMK | IL2RG | PPP1R12A | JUND |
| FCER1G | LBH | TMA7 | RPS2 |
| TYROBP | SLFN5 | IER2 | RPS4X |
| CST7 | RPS15A | RPLP1 | HSPA1A |
| IFITM1 | CLEC2B | RPS23 | JUN |
| SAMD3 | GSTP1 | EOMES | IQGAP1 |
| CCL4 | RPS27A | STK17B | RPS8 |
| SH2D1A | DUSP2 | RPL19 | FYB |
| PRF1 | BTG1 | MYL12A | RPL6 |
| PVRIG | AKNA | CD52 | ITGB2 |
| TARP | PYHIN1 | ID2 | NR4A2 |
| APOBEC3G | RUNX3 | RPS9 | FAU |
| RAC2 | RPL41 | RPS3 | DDX5 |
| GLIPR2 | TC2N | TPT1 | H3F3B |
| FGR | RPS29 | ACTG1 | GNG2 |
| GNLY | FOSB | RPL18A | RPS24 |
| PTPRCAP | RPS14 | RPL34 | NEAT1 |
| PTPRC | PFN1 | PABPC1 | TXNIP |
| CD69 | CCL3 | RPS19 | ABCB1 |
| LCP1 | TTN | FOS | DUSP1 |
| PTPN22 | EVI2B | MS4A1 | RPL30 |
| GIMAP7 | CD96 | RPL26 | RPS25 |
| PRKCH |  |  |  |

**Table S3** Discrimination of the HNK-10 model and other clinical indicators

|  | NRI (95%CI) | IDI (95%CI) |
| --- | --- | --- |
| HNK-10 | - | - |
| Grade | 0.30 (0.159-0.446) | 0.09 (0.006-0.127) |
| Stage | 0.08 (0.075-0.229) | 0.03 (0.008-0.069) |
| Age | 0.27 (0.116-.0418) | 0.09 (0.050-0.121) |
| Gender | 0.17 (0.012-0.334) | 0.08 (0.046-0.118) |

CI, confidence interval; IDI, integrated discrimination improvement; NRI, net reclassification improvement. NRI or IDI>0 indicated the new model (HNK-10) had better prediction performance than other clinical indicators. cut-off of NRI: 0.2, 0.4.

**Supplementary Figure**


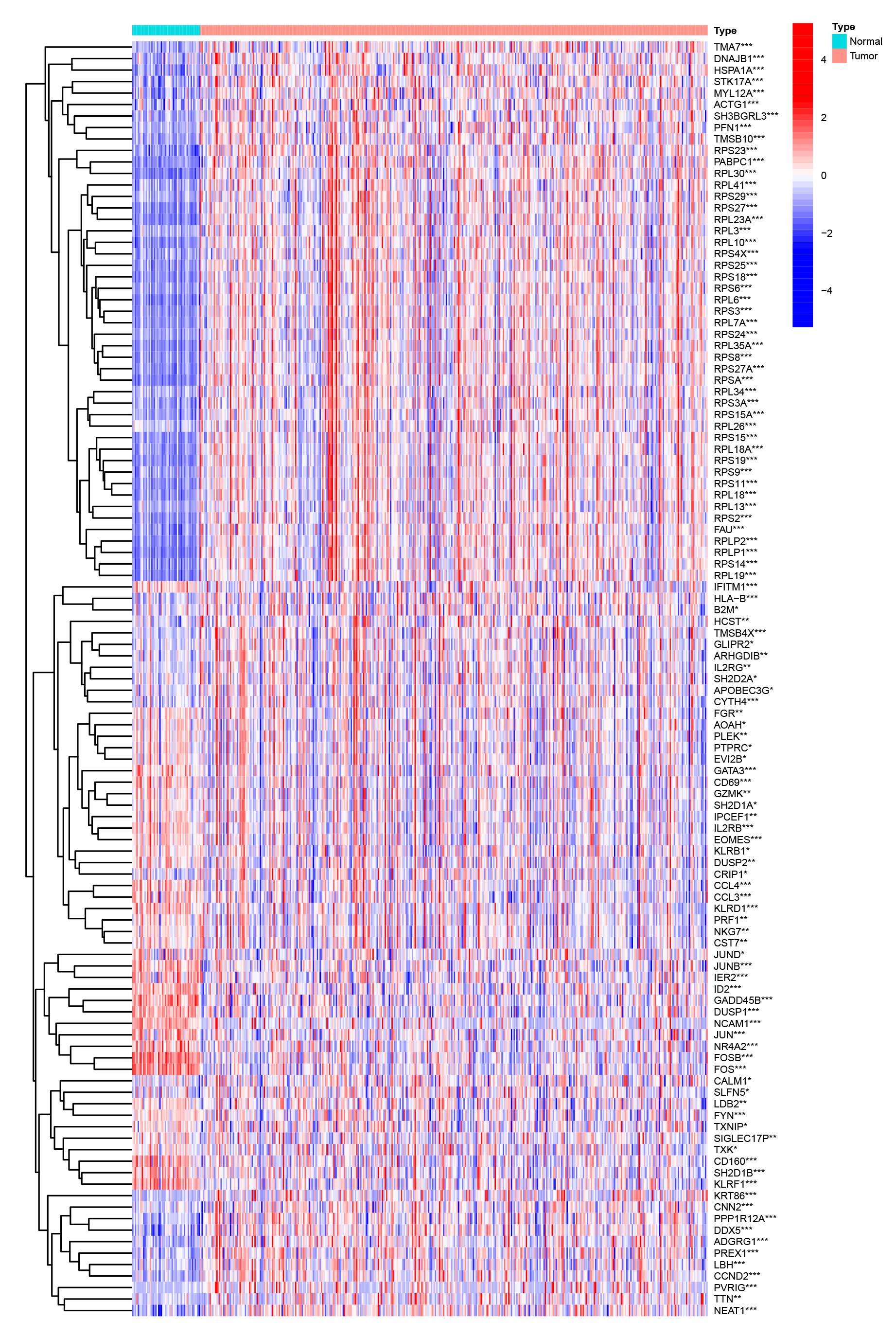


**Figure S1** The heatmap of differential NKMGs expression between tumor and normal tissues.


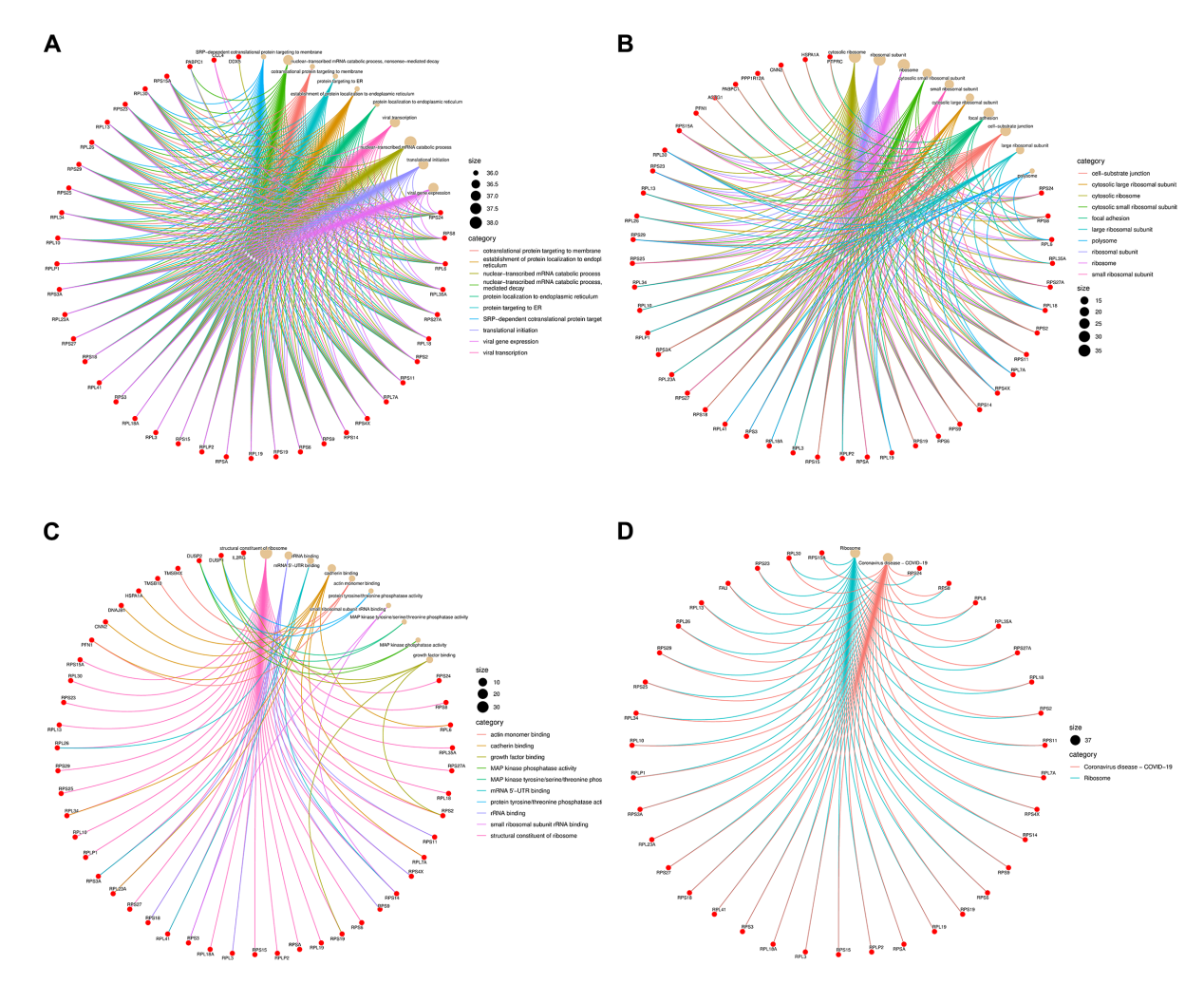


**Figure S2** GO pathway enrichment results in (A) biological process (BP), (B)cellular component (CC), and (C)molecular function (MF). (D)KEGG pathway enrichment results.

**
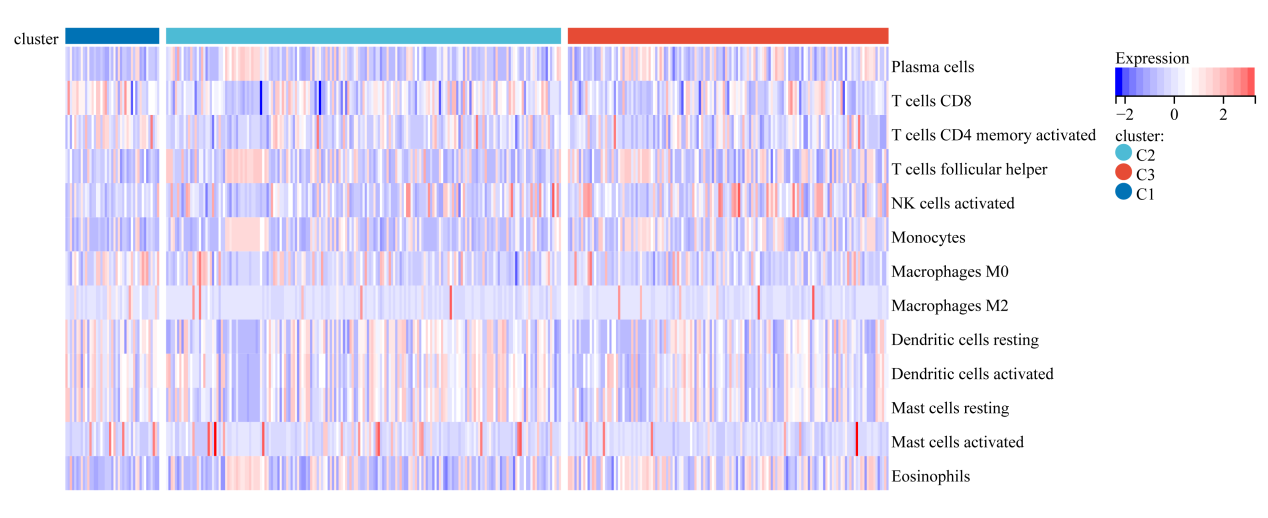
**

**Figure S3** The heatmap of immune infiltration cells between different risk groups.


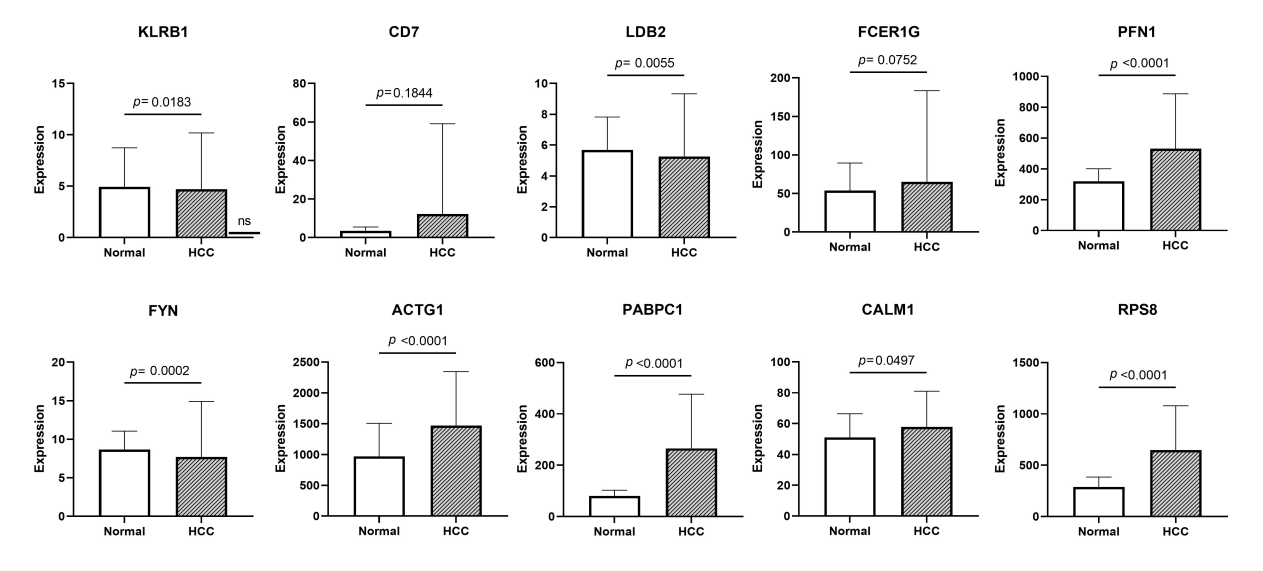


**Figure S4** The expression levels of 10 prognosis genes in the TCGA dataset.


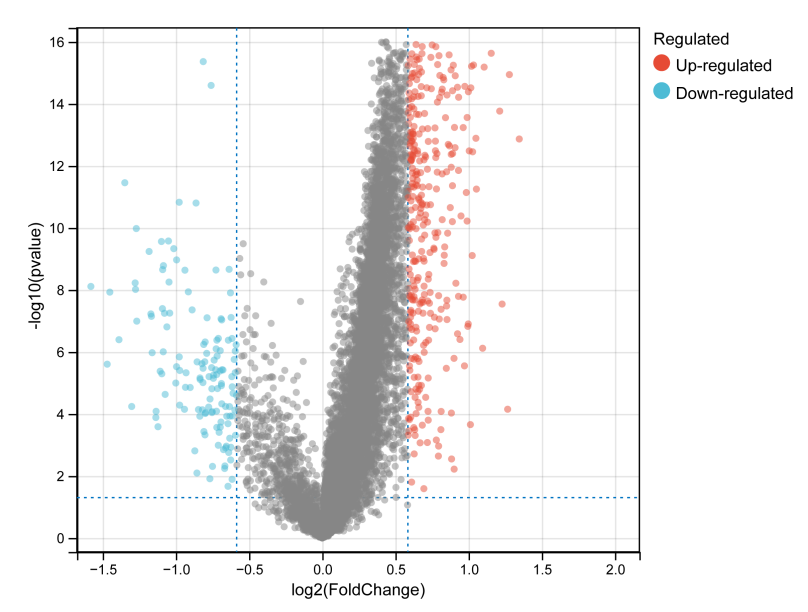


**Figure S5** Volcano plot exhibiting up-regulated and down-regulated genes in different risk groups.

**
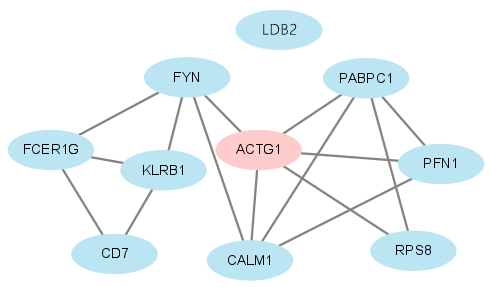
**

**Figure S6** PPI analysis of 10 prognosis genes and the hub gene ACTG1.
